# The global burden of neglected zoonotic diseases: current state of evidence

**DOI:** 10.1093/eurpub/ckac129.757

**Published:** 2022-10-25

**Authors:** C di Bari, N Venkateswaran, D Pigott, C Flastl, B Devleesschauwer

**Affiliations:** 1 Epidemiology and Public Health, Sciensano, Brussels, Belgium; 2 Department of Health Metrics Sciences, Institute for Health Metrics and Evaluation, Seattle, USA; 3 Department of Translational Physiology, Infectiology and Public Health, Ghent University, Ghent, Belgium

## Abstract

The majority of emerging infectious diseases are zoonoses, most of which are classified as “neglected”. By affecting both humans and animals, zoonoses pose a dual burden. The disability-adjusted life year (DALY) metric quantifies human health burden using mortality and morbidity. This review aims to describe and analyze the current state of evidence on the burden of neglected zoonotic diseases (NZDs) and start a discussion on the current understanding of the global burden of NZDs. We identified 26 priority NZDs through consulting the CDC One Health Zoonotic Disease Prioritization Exercise, the Joint External Evaluation reports, and the WHO roadmap for NTDs. A systematic review of global and national burden of disease (BoD) studies for these priority NZDs was conducted using pre-selected databases. Data on diseases, location and DALYs were extracted for each eligible study. A total of 1887 records were screened, resulting in 72 eligible studies (58 national or sub-national, 12 global, and 2 regional studies). The highest number of BoD studies was found for non-typhoidal salmonellosis (23), whereas no estimates were found for West Nile, Marburg and Lassa fever. Geographically, the highest number of studies were found in the Netherlands (11), China (5) and Iran (4). The number of BoD studies retrieved mismatched the perceived importance in national prioritization exercises. For example, anthrax was considered a priority NZD in 73 countries, but only one national estimate was retrieved. By summing the available global estimates, these diseases would cause at least 10 million DALYs in total. The burden of NZDs at the global level remains scattered, and trends were challenging to identify. There are several priority NZDs for which no burden estimates exist, and the number of BoD studies does not reflect national disease priorities. To have complete and consistent estimates of the global burden of NZDs, these diseases should be integrated into larger global BoD initiatives.

**Key messages:**

• There is a mismatched between the estimated retrieved in the search and the perception of the importance of these disease. This amplify the need for a comprehensive program.

• No complete list of zoonoses exist, and the definition used is vague. A stricter definition of zoonoses and what defines them will help provide a clear view of dealing with and controlling them.

